# The olivo-cerebellar system: a key to understanding the functional significance of intrinsic oscillatory brain properties

**DOI:** 10.3389/fncir.2013.00096

**Published:** 2014-01-28

**Authors:** Rodolfo R. Llinás

**Affiliations:** Department of Physiology and Neuroscience, New York University School of MedicineNew York, NY, USA

**Keywords:** intrinsic oscillatory, olivo-cerebellar, electrophysiology, IO neurons, PO neuron oscillation

## Abstract

The reflexological view of brain function (Sherrington, 1906) has played a crucial role in defining both the nature of connectivity and the role of the synaptic interactions among neuronal circuits. One implicit assumption of this view, however, has been that CNS function is fundamentally driven by sensory input. This view was questioned as early as the beginning of the last century when a possible role for intrinsic activity in CNS function was proposed by Thomas Graham Brow (Brown, 1911, 1914). However, little progress was made in addressing intrinsic neuronal properties in vertebrates until the discovery of calcium conductances in vertebrate central neurons leading dendritic electroresponsiveness (Llinás and Hess, 1976; Llinás and Sugimori, 1980a,b) and subthreshold neuronal oscillation in mammalian inferior olive (IO) neurons (Llinás and Yarom, 1981a,b). This happened in parallel with a similar set of findings concerning invertebrate neuronal system (Marder and Bucher, 2001). The generalization into a more global view of intrinsic rhythmicity, at forebrain level, occurred initially with the demonstration that the thalamus has similar oscillatory properties (Llinás and Jahnsen, 1982) and the ionic properties responsible for some oscillatory activity were, in fact, similar to those in the IO (Jahnsen and Llinás, 1984; Llinás, 1988). Thus, lending support to the view that not only motricity, but cognitive properties, are organized as coherent oscillatory states (Pare et al., 1992; Singer, 1993; Hardcastle, 1997; Llinás et al., 1998; Varela et al., 2001).

## Introduction

The reflexological view of brain function (Sherrington, 1906) has played a crucial role in defining both the nature of connectivity and the role of the synaptic interactions among neuronal circuits. One implicit assumption of this view, however, has been that CNS function is fundamentally driven by sensory input.

This view was questioned as early as the beginning of the last century when Thomas Graham Brown proposed a possible role for intrinsic activity in CNS function (Brown, 1911, 1914). However, little progress was made in addressing intrinsic neuronal properties in vertebrates until the discovery of calcium conductances in vertebrate central neurons leading dendritic electroresponsiveness (Llinás and Hess, 1976; Llinás and Sugimori, 1980a,b) and subthreshold neuronal oscillation in mammalian inferior olive (IO) neurons (Llinás and Yarom, 1981a,b). This happened in parallel with a similar set of findings concerning invertebrate neuronal system (Marder and Bucher, 2001). The generalization into a more global view of intrinsic rhythmicity at forebrain level occurred initially with the demonstration that the thalamus has similar oscillatory properties (Llinás and Jahnsen, 1982) and the ionic properties responsible for some oscillatory activity were, in fact, similar to those in the IO (Jahnsen and Llinás, 1984; Llinás, 1988). Thus, lending support to the view that not only motricity, but also cognitive properties, are organized as coherent oscillatory states (Pare et al., 1992; Singer, 1993; Hardcastle, 1997; Llinás et al., 1998; Varela et al., 2001).

Concerning the functional significance of IO intrinsic properties two main issues should be addressed; (1) the predictive aspects movement intentionality and its translation into motor strategy and tactics and (2) the timing of motor execution. In reviewing the global properties of IO function I will briefly address general anatomy and electrophysiology of IO nucleus and it neurons.

### General IO anatomy

**Figure 1:**
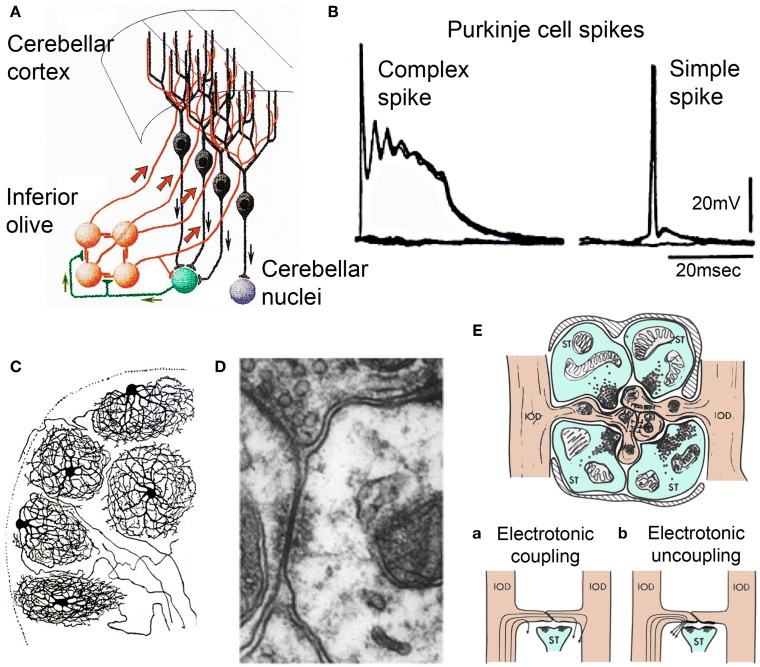
**Olivocerebellar circuit**. **(A)** Cerebellar cortex. Inferior olive axons (IO, orange) activate Purkinje cells (black) through the climbing fibers and send collaterals to cerebellar nuclear cells (green) that feed back to IO and projection cerebellar nuclear cells (purple) (After Ramon y Cajal, 1911). **(B)** Intracellular recordings of an-all or-none complex spike elicited by climbing fiber stimulation and a simple spike elicited by mossy fiber activation. **(C)** Inferior olivary neurons (Ramon y Cajal, 1911). Note the spherical dendritic trees. **(D)** Electronmicrograph showing gap junction between spines of IO dendrites within IO glomerulus. Modified from Llinás et al. (1974). **(E)** Diagram of IO glomerulus. The center shows spines from IO dendrites (IODs) coupled by gap junctions. (a) coupling current path between IO dendrites, (b) current flow shunted when gabaergic synapses are active at the gap junction. (Modified from Llinás, 1974).

The olivocerebellar system is one of the most conserved in the vertebrate brain, being present in all such forms studied (Ariens-Kappers et al., 1936). It comprises a set of bilaterally symmetrical inferior olivary nuclei (IO) and the overlaying cerebellum. These two structures are mutually linked through axonal pathways within the cerebellar peduncles. Some IO neurons have spherical dendritic trees (Figure 1C). Their axons traverse the midline at the bulbar region (Figure 1A, orange), course up the contralateral cerebellar peduncle, and enter the cerebellar white matter. From there branches establish excitatory synaptic contacts with the cerebellar nuclear neurons (Figure 1A green and purple) while the axons proceed into the cerebellar cortex to establish the most powerful synaptic contact in the brain the so called climbing fiber Purkinje cell synapse (Ramon y Cajal, 1911) (Figure 1A, black). This synapse is a one-to-one chemical junction and is all of IO origin, exclusively (Szentagothai and Rajkovits, 1959). This input establishing hundreds of junctions with the large spines in the main branches of the Purkinje cell dendritic tree. Activation of a climbing fiber elicits an all-or-none excitatory response in the Purkinje cells (Eccles et al., 1965, 1966a,b,c) (Figure 1B, left trace) later named a “complex spike,” (Thach, 1968) as opposed to the simple spike produced by parallel fiber activation (Figure 1B, right trace). There are about ten times more PCs than IO neurons and so each IO neuron generates an average of ten climbing fibers (Armstrong and Schild, 1970). The PC axons, the only output of the cerebellar cortex, terminate in the cerebellar and related vestibular nuclei where they form inhibitory synapses (Ito and Yoshida, 1966) (Figure 1A). Cerebellar nuclei neurons are the only output of the cerebellum.

Concerning the cerebellar nucleus neurons they exist in two varieties with about half being excitatory and the other half inhibitory. The excitatory variety innervates brain stem, thalamus, and spinal cord via direct and indirect pathways. The inhibitory neurons return, in their entirety, to the centro-lateral IO where they form synapses in structures called “glomeruli” (Figure 1E) as well as with the dendritic tree directly (Sotelo et al., 1986; de Zeeuw et al., 1990a; Fredette and Mugnaini, 1991; Medina et al., 2002). Each IO glomerulus contains five to eight spines from dendrites of different IO neurons and support IO electrotonic coupling via gap junctions (Llinás, 1974; Llinás et al., 1974; Sotelo et al., 1974; de Zeeuw et al., 1990b) (Figure 1D). The degree of coupling is, thus, dynamically modulated by the inhibitory synaptic shunting (Figure 1E) (Llinás, 1974; Lang et al., 1996) as a feed back from the cerebellar nuclear output (de Zeeuw et al., 1990a, 1996).

## Timing P rope R ties of the olivocerebellar system

### Motor coordination and timing

**Figure 2:**
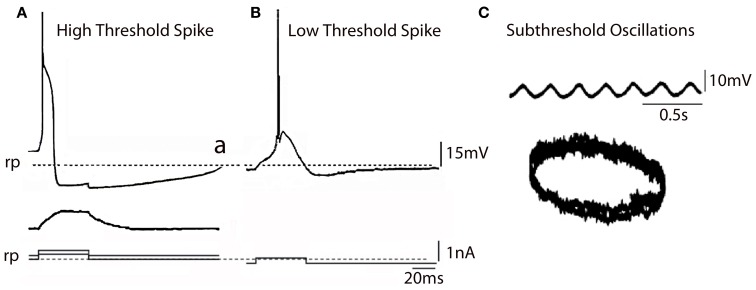
**IO Electrophysiology. (A)** *In vitro* intracellular recordings from IO neuron showing high threshold spike (a) activated by an outward pulse from a depolarized potential with respect to rest (broken line) the same outward pulse delivered from the rest potential (broken line) did not elicit a spike (b). **(B)** Same pulse as in **(A)** delivered from a hyperpolarized membrane potential level generated a low voltage activate spike (Modified from Llinás and Yarom, 1981a,b). **(C)** Subthreshold membrane oscillation recorded intracellularly from an IO neuron and associated *Lissajeux* image demonstrating oscillatory stability. (Modified from Llinás and Yarom, 1986). rp, resting potential.

**Figure 3:**
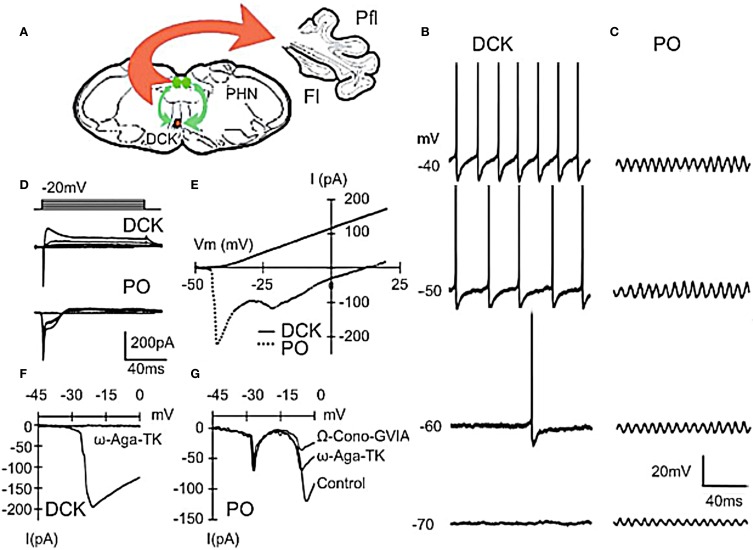
**Differences between principal olive (PO) and Dorsal cap of Kooy (DCK) neurons. (A)** Drawing lf brain slice showing climbing fiber projection (red) of DCK neurons to the contralateral cerebellar flocculus, and afferent GABAergic input (green) to the DCK from the bilateral prepositus hypoglossi nuclei. **(B–D)** Effect of membrane potential on DCK and PO firing (in same slice).** (B)** Recordings from DCK neuron showing increased firing frequency with membrane potential. Each action potential is followed by a large, long-lasting afterhyperpolarization. (Action potentials were truncated and recorded with a high-potassium intracellular solution) **(C)** Patch recordings from a PO neuron showing subthreshold oscillations at the same membrane potentials as in **(A)** Amplitude increased, but frequency was unchanged. **(D)** Representative currents in DCK and PO neurons (in response to 100-ms depolarizing square pulses recorded by using a high-potassium electrode solution). Inward currents were followed by a small outward current in PO neurons while the same depolarizing square pulses activated a strong outward current in DCK neurons. **(E)** I–V curve for cells in panel **(D)**. **(F)** DCK neurons had a single current peak near a membrane potential of −20 mV that was blocked by ω-Agatoxin-TK (a specific P/Q calcium channel blocker). **(G)** In the PO cells, two inward components, peaking near −20 and −10 mV were seen. The second component was reduced by application of ω-Agatoxin-TK and further reduced by application of ω-Conotoxin-GVIA (a specific N-type calcium channel blocker). Thus, the inward calcium currents of DCK neurons were mediated only by P/Q-type channels, while both P/Q-type and N-type channels are present in PO neurons. Fl, flocc; Pfl, paraflocculus; PHN, prepositus hypoglossi nuclei. (Modified from Urbano et al., 2006).

**Figure 4:**
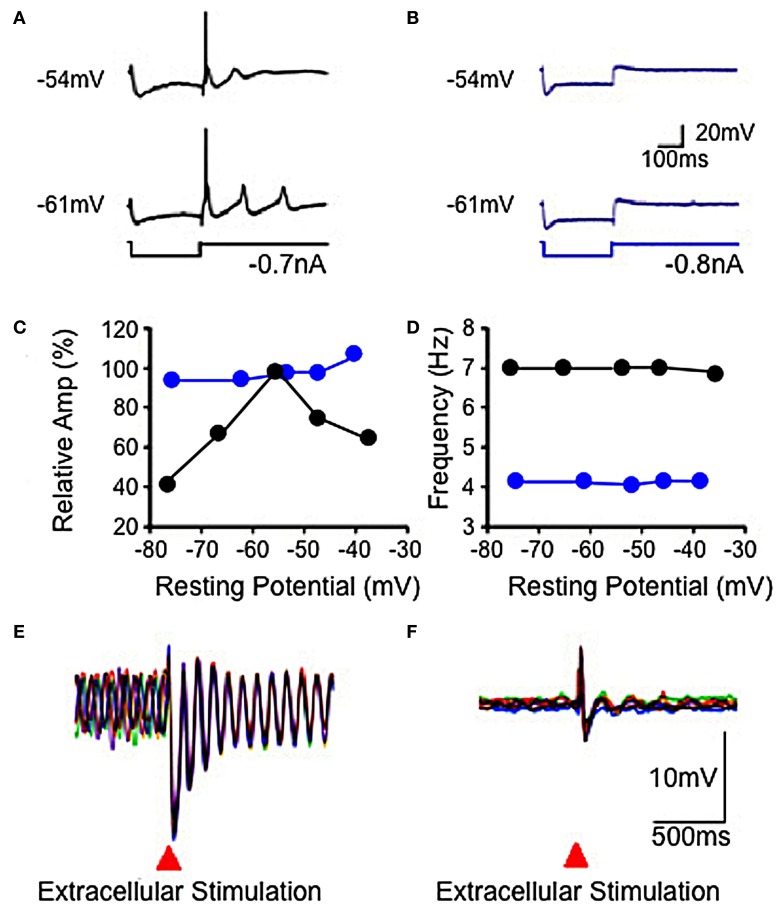
**Electrophysiological properties of IO in wild-type and mutant mice. (A,B)** Hyperpolarizing current injection elicited a low threshold spike from IO cell in slice from wild-type mouse **(A)**, but not from mutant mouse **(B)** at resting potentials of −54 and −61 mV. Subthreshold rebound mediated by Ih was present in the mutant mouse. **(C)** Plot showing modulation of subthreshold sinusoidal oscillation (SSTO) amplitude by membrane potential in wild-type (black) but not in mutant (blue) mice. **(D)** Frequency of SSTO was lower in mutant than in wild-type mice but neither was modulated by membrane potential. **(E,F)** Superposition of six traces showing SSTO recorded from single IO neuron in wild-type **(E)** or mutant **(F)** mouse. Extracellular stimulation lead to phase reset of SSTO in IO cell in slice from the wild-type mouse. Such stimulation had a minor, if any, effect in the mutant mouse **(F)**. (Modified from Choi et al., 2010).

**Figure 5:**
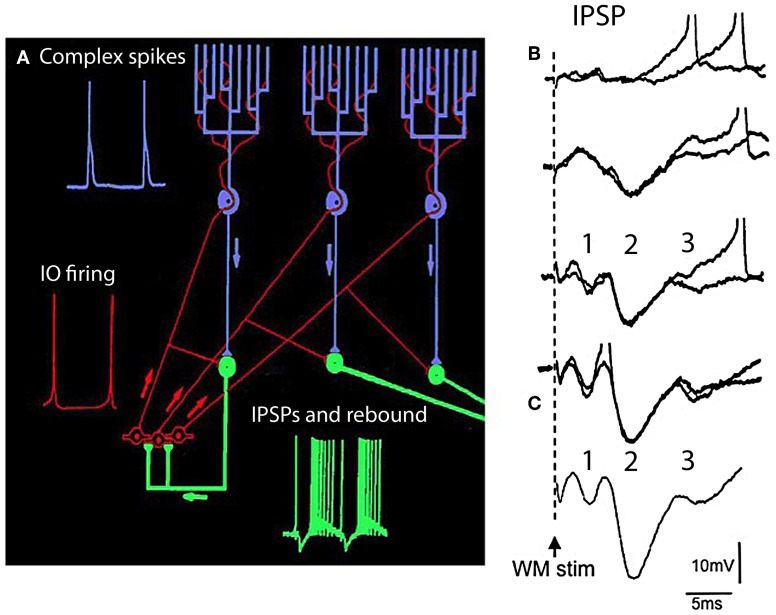
**The olivocerebellar loop circuit. (A)** Diagram of olivocerebellar circuit. Action potentials in IO neurons (red) are generated at the crest of the subthreshold oscillations; example of subthreshold oscillations is shown in Figure 2C. These elicit complex spikes in Purkinje cells (green) and activate cerebellar nuclear cells (purple and yellow). Purkinje cell output is inhibitory to cerebellar nuclear cells where the IPSPs trigger rebound firing in cerebellar nuclear cells. Arrows indicate direction of action potential conduction.** (B,C)** Synaptic potentials and firing of cerebellar nuclear cells. White matter stimulation (WM stim) at increasing stimulus strength elicits graded EPSP-IPSP sequences. The first sequence (1) is due to direct stimulation of mossy fiber collaterals (EPSP) and Purkinje cell axons (IPSP). The second sequence is due to activation of the climbing fiber system (2) the Purkinje cell IPSP was strong enough to activate the rebound response (3 and spikes). **(C)** Average of 10 responses showing the timing of the EPSP-IPSP sequences. (Modified from Llinás and Muhlethaler, 1988).

**Figure 6:**
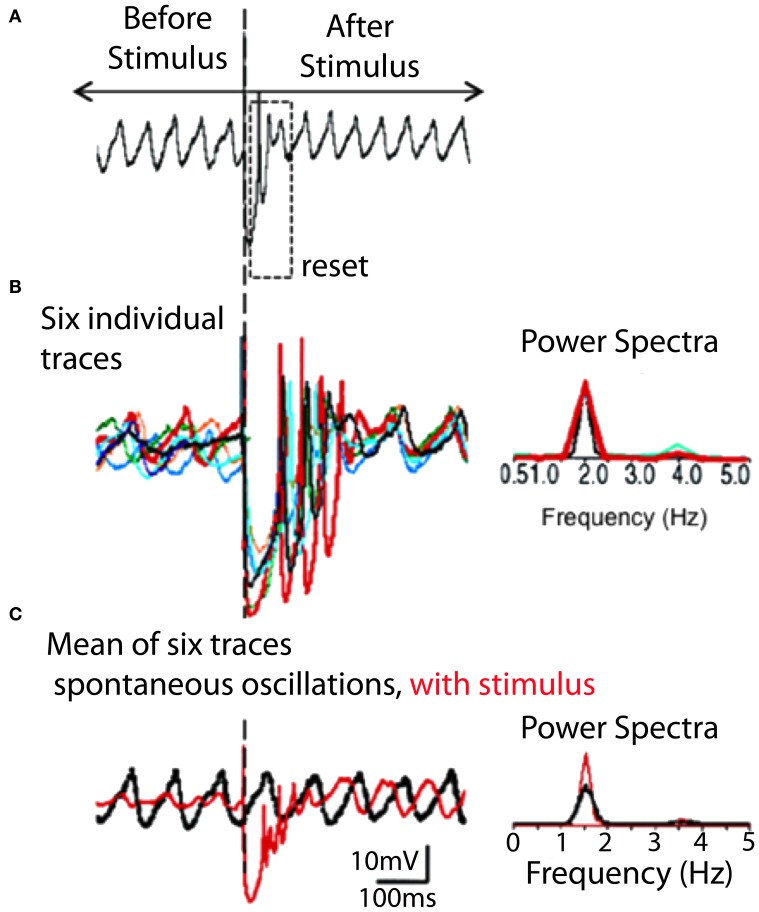
**IO spontaneous and stimulus-evoked oscillations**. **(A)** Intracellular recording of spontaneous oscillations at 2 Hz interrupted by an extracellular stimulus. After extracellular stimulation the oscillations disappeared for 750 ms (boxed area) and then resumed. **(B)** Left. Superimposition of six individual intracellular traces (each a different color) of stimulus-evoked oscillations recorded from the same cell. Right. Power spectra. The frequency of stimulation-evoked oscillation was the same (2.0 Hz). Oscillations are clear after the stimulus-induced reset but can be barely detected before the stimulation. **(C)** Superposition of average of six traces of stimulus-evoked oscillations (red) and spontaneous oscillations (black). The stimulus-evoked and spontaneous oscillations have the same frequency. Calibration, 1 mV; **(A)** 1 s; **(B)** 415 ms; **(C)** 500 ms. (Modified from Leznik et al., 2002).

**Figure 7:**
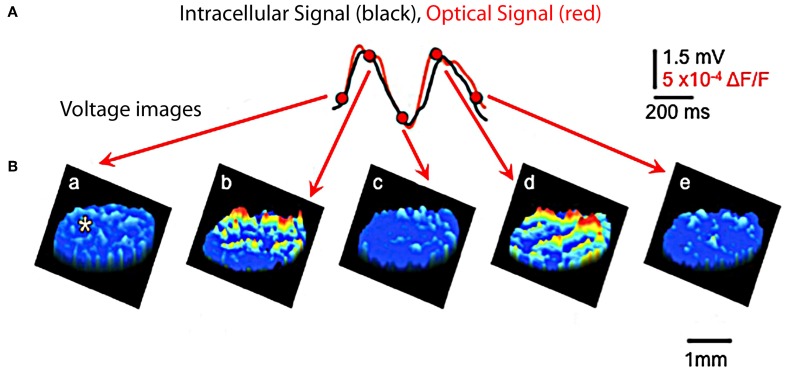
**Optical and intracellular recordings in brainstem slice. (A)** Recordings of IO subthreshold oscillations are quite similar using optical (red) and using intracellular voltage (black) methods. (Recording site indicated by asterisk in first image panel in **B**). **(B)** Spatial profiles of optically recorded oscillations at five times during the oscillation shown in panel **(A)**. (Modified from Leznik and Llinás, 2005).

**Figure 8:**
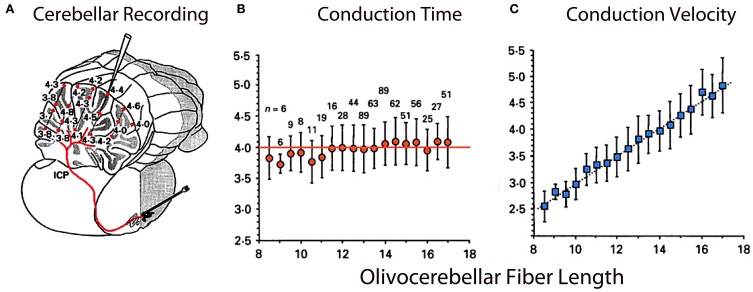
**Olivocerebellar conduction times. (A)** Drawing of cerebellum showing conduction times for evoked complex spikes (parasagittal plane 4 mm from midline) in an *in vivo* preparation. **(B)** Plot of mean conduction time (±sem) as a function of olivocerebellar fiber length (0.5 mm bins) showing that conduction time was independent of distance between IO and individual Purkinje cell. **(C)** Plot showing close to linear relationship between mean conduction velocity (±sem) and fiber length. (Modified from Sugihara et al., 1993).

Concerning motor coordination and timing three general issues are evident in the electrophysiology of the olivocerebellar system. (1) The system generates a timing signal that is inscribed in the intrinsic electrical properties of single IO (Figures 2–4) and cerebellar nuclear (Figure 5) neurons, (2) the organization of the nucleus via electrical coupling allows for synchronous multicellular temporal coherence that generates a close to simultaneous neuronal cluster activation (Figures 6, 7), and (3) due to the remarkable property of conduction isochronicity (Figure 8) the timing signal does not disperse against distance as it is conducted along the pathways carrying it to the final integration sites at cerebellar nuclear level. Each of these issues will be considered in turn.

### Single cell electrophysiology

With the exception described below, concerning two types of IO neurons, most of the olivocerebellar has been known to generate synchronous rhythmic activity attributed to the intrinsic oscillatory properties of the IO neurons (Llinás and Yarom, 1981a,b; Benardo and Foster, 1986; Bal and McCormick, 1997) and their multicellular synchrony supported by their electrotonic coupling (Llinás, 1974; Sotelo et al., 1974; Llinás and Yarom, 1981a,b; Lampl and Yarom, 1997; Makarenko and Llinás, 1998; Yarom and Cohen, 2002). Recently, asynchronous release of GABA has been reported to determine an inhibitory regulation of electrical coupling of neurons in the IO (Best and Regehr, 2009).

Such intrinsic oscillatory properties are supported by a set of voltage-dependent calcium and potassium conductances (in addition to those involved in action potential generation) enabling IO cells to oscillate and fire rhythmically at 1–10 Hz. These conductances include a high-threshold Ca2+ conductance, a low-threshold Ca2+ conductance, a Ca2+-activated K+ conductance, and a hyperpolarization-activated cationic conductance (Llinás and Yarom, 1981a,b, 1986; Bal and McCormick, 1997). There is as mentioned above, a separate group of IO neurons with quite different electrical properties.

#### Two basic IO neuron types

An important, but often forgotten, aspect of IO function is the fundamental differences between two parts of the olive, what may be called the principal olive (PO) represented by the three main nuclei (lateral medial and central) and the Dorsal Cap of Kooy (DCK). Morphologically PO neurons are characterized by a spherical dendritic tree (Figure 1C) while the dendrites of DCK neurons have a bipolar-like arrangement that extended farther away from the soma (Urbano et al., 2006). The two different morphological type neurons present in the IO are also electrophysiologically distinct.

The PO comprises most of the IO neurons, are concerned with limb and digit movements and to the so-called “physiological tremor” i.e., the non-continuous nature of motor organization (Llinás, 1991; Welsh and Llinás, 1997). This tremor supports the timing of motor execution in all systems other than the oculomotor. The functional character of PO neurons can be easily observed by measuring, for instance, the velocity of voluntary human finger movements (Vallbo and Wessberg, 1993) that occur at a close to constant of 8–10 Hz steps independently of movement speed. Synchronous IO oscillations have been shown to modulate periodic vibrissal movements (Lang et al., 2006) in the same frequency range. Electrophysiologically, such IO neurons are characterized by their ability to generate high-threshold Figure 2A and “low-threshold” calcium spike as recorded *in vitro* Figure 2B (Llinás and Yarom, 1981a,b). The latter is generated by the activation of both T-type calcium channels (Cav3.1) and Ih potassium currents (Llinás and Yarom, 1981a,b, 1986; Bal and McCormick, 1997; Lampl and Yarom, 1997), which limits their frequency to 8–10 Hz. PO neurons are characterized by subthreshold oscillations at 8–10 Hz that are very stable (Figure 2C). *In vivo* intracellular recordings from IO neurons have shown transient subthreshold oscillations at 6–12 Hz with spikes generated on the depolarization phase of the oscillations (Chorev et al., 2007). A single action potential in the IO triggers a burst of axonic spikes. The properties of the spike burst are modulated by the phase (Mathy et al., 2009) and or amplitude (Bazzigaluppi et al., 2012) of the subthreshold oscillations.

The DCK is a smaller nucleus that is involved with the organization of eye movements. These neurons lack both T-type calcium channels and the Ih potassium current (Urbano et al., 2006) underlying the intrinsic subthreshold oscillation characteristics of PO neurons and so do not display subthreshold oscillatory behavior. They are, however, electrotonically coupled, but only to each other, shunning contacts with their oscillatory counterpart (Urbano et al., 2006). These fundamental differences go a long way toward addressing the apparent functional inconsistencies that have plagued the field of cerebellar motor control and, more importantly, give further support to the findings concerning the time binding proposal for non-ocular motricity (Carpenter, 1977; Farmer, 1998; Llinás, 2009).

Because DKC Neurons do not oscillate one of the arguments often voiced against the timing hypothesis of IO function has related to the absence of the physiological tremor in the oculomotor system (Carpenter, 1977). Thus, as stated above, while physiological tremor is observed in somatomotor systems (Llinás, 1991; Vallbo and Wessberg, 1993; Lang et al., 2006), where it has been shown to play an important role in motor binding by providing coherent activation of the motoneuronal pools responsible for motor execution, such physiological tremor is conspicuously absent in ocular motricity. Indeed, it has been known for many years now (Carpenter, 1977) that the oculomotor system is capable of both smooth pursuit (an object is followed on a moving trajectory) and saccadic eye movements (the eye position is quickly reset having reached maximal displacement from its central orbital position).

The findings of a recent study comparing the electrophysiological properties of PO and DCK neurons helps explain the discrepancies observed between somato-motor and oculomotor cerebellar control (Figure 3). DCK neurons, identified using Biocytin during patch recordings (Urbano et al., 2006), responded differently to current injection than do PO cells. They did not present an h-current- dependent “depolarizing sag” during hyperpolarization and the T-current-dependent rebound of membrane potential was absent. When depolarized, DCK fired at a much higher frequency than PO neurons. The average frequency of DCK firing could reach gamma-band frequencies (>30 Hz) while PO neurons only reached theta-band range (4–8 Hz). The same frequency range of membrane potential events was observed using voltage sensitive dye imaging. We stained transversal slices using the voltage-sensitive dye di-4ANEPPS and used a bipolar electrode to deliver a pair of stimuli (50 Hz, 2 shocks of 200 μs of duration) at the edge of the DCK nucleus. After such stimulation the entire DCK nucleus depolarized rhythmically with peaks of activity every 1.5 s.

#### PO neuron oscillation are dynamically regulated by P/Q-type and T-type calcium channels

Concerning mechanisms responsible for membrane potential oscillation in PO neurons, one of the remarkable properties is the set of ionic conductances that generate such electrical activity. Thus, the electrophysiological properties of IO neurons have recently been investigated using knock out (KO) mice that lacked the gene for the pore-forming α1G subunit of the T-type calcium channel (CaV3.1^−/−^) and their littermate wild type (WT) mice (Choi et al., 2010). The low-threshold calcium spike and the sustained endogenous oscillation following rebound potentials were absent in IO neurons from CaV3.1^−/−^ mice.

In addition to spikes, PO neurons support spontaneous subthreshold membrane potential oscillations near 10 Hz (see Figure 2C) (Benardo and Foster, 1986; Llinás and Yarom, 1986). It has been proposed that calcium current and calcium-activated potassium current may be account for the oscillatory behaviors of IO neurons (Llinás and Yarom, 1981a,b, 1986). The other group of mice lacked the gene for the pore-forming α1G subunit of the T-type calcium channel (CaV3.1^−/−^). In these mice the LTS, activated as a rebound from a hyperpolarizing square current pulse, was absent (compare Figures 4A,B) but the HTS was not affected. Although the rebound activity mediated by the hyperpolarization-activated cation current (Ih) was still present in IO neurons from CaV3.1^−/−^ mice, it was not strong enough to evoke sodium spikes (Figure 4B). IO neurons from these mice also showed altered patterns of subthreshold oscillations and the probability of their occurring was only 15%, significantly lower than the one found in wild-type animals (78%). In addition, the low-threshold calcium spike and the sustained endogenous oscillation of rebound potentials were absent in IO neurons from these mice. The results from studies of these KO mice suggest that both α1A P/Q- and α1G T-type calcium channels are required for the dynamic control of IO oscillations.

No significant changes in the input resistance, time constant, and capacitance of membrane were observed between IO neurons recorded in either mutants or WT mice (Choi et al., 2010). These findings indicate that the α1A P/Q-type calcium channels are involved in the generation of HTS and calcium conductance by α1G T-type calcium channels also play a crucial role in the generation of LTS.

In WT mice IO cell oscillation modulate spike initiation, and so action potentials are normally generated at the crest of such oscillations and fire at 1–10 Hz. This intrinsic rhythm is thus entrained with the speed of movement execution as mentioned above. Moreover, the phase of subthreshold oscillations may be influenced by subthreshold activity, as shown in Figures 4E,F). Indeed, extracellular local electrical stimuli, or strong excitatory synaptic input will reset the phase, but not the amplitude or frequency, of subthreshold oscillations (Leznik et al., 2002).

### Electrotonic coupling

Concerning the electrical coupling, as in other CNS structures (Bennett, 2000), gap junctions constitute the main communication pathway between the IO neurons (Sotelo et al., 1974; de Zeeuw et al., 1996; Devor and Yarom, 2002). Such electrotonic coupling has been assumed to play a crucial role in synchronizing IO oscillations and in generating groups of concurrently oscillating neurons (Llinás and Yarom, 1986). This coupling was also assumed to be controlled by return glomerular inhibition (Llinás, 1975). IO afferents were, in fact, found to modulate the efficiency of electrotonic coupling via inhibition at the glomerulus. The pathway function is actually supported, as stated above, by the cerebellar nuclear GABAergic neurons (Sotelo et al., 1986; Fredette and Mugnaini, 1991; de Zeeuw et al., 1996; Medina et al., 2002). These neurons represent 50% of the total neuronal population in such nuclei giving some measure of the importance of this feedback inhibitory pathway. Accordingly, it was determined that such input can control the degree and distribution of synchronous oscillatory activity in the IO nucleus (Leznik and Llinás, 2005) and the cerebellar cortex (Lang et al., 1996; Lang, 2001, 2002). Moreover, dynamic groups of IO neurons oscillating in-phase can synchronously activate a population of PCs and thereby control patterns of synchronous activity in the cerebellum during motor coordination (Welsh et al., 1995). Models of IO cells that includes conductances as well as gap junctions explores the interaction of coupling strength, membrane potential level, and conductance modulation in IO synchronization at the network level (Manor et al., 1997; Schweighofer et al., 1999, 2004a,b; Manor et al., 2000; Jacobson et al., 2008; Torben-Nielsen et al., 2012) and effect on the climbing fiber burst (De Gruijl et al., 2012).

As in other brain regions the gap junctions are formed by connexin 36 (Cx36) (Condorelli et al, 1998; Belluardo et al., 2000; Rash et al., 2001). Yet, in Cx36 knock-out mutant mice subthreshold oscillations are present (Long et al., 2002). This has been shown to be due to morphological and electrophysiological compensations in the mutant IO neurons making them more excitable (De Zeeuw et al., 2003). A recent study utilizing tracers and paired electrophysiological recordings has shown that the coupling between IO neurons is highly variable (Hoge et al., 2011). This introduces another important parameter in considering IO function in motricity.

#### Visualization of IO cluster activity

Although synchronized IO oscillations are a neuronal ensemble event, they have been studied primarily on a single-cell level and no information has been available about their spatial profiles. Thus, an attempt was made to address this issue by utilizing voltage-sensitive dye optical imaging (Leznik and Llinás, 2005; Leznik et al., 2002). This technique is presently the methodology of choice in studying the geometrical distribution of activity in a large neuronal ensemble (see, for instance, Ebner and Chen, 1995). We have shown that ensemble oscillations in the IO originate in synchronized activity clusters, where each cluster is a localized functional event composed of hundreds of cells. Given the distribution of complex spike activity in the cerebellum cortex, we have proposed that these clusters are very likely to be responsible for the synchronized activation of the PCs observed in previous *in vivo* multielectrode experiments (Lang et al., 1996). Furthermore, when comparing our experimental results with those obtained by computational modeling of IO neuronal ensembles endowed with oscillatory electrical properties and electrotonic coupling (Makarenko and Llinás, 1998; Velarde et al., 2002), we could show that neuronal oscillatory clustering is a direct consequence of the combined electrotonic/intrinsic properties of coupled IO neurons (Leznik et al., 2002).

While electrical recording of IO neurons *in vitro* had indicated the possibility that electrically coupled IO cells could actually cluster into synchronized ensemble neuronal groupings, there was no direct demonstration of such dissipative structures. In searching for such dissipative structures, voltage-sensitive dye imaging of oscillatory activity was attempted and successfully implemented in rodent IO slices (Leznik et al., 2002). Thus, spatio-temporal profiles of ensemble IO oscillations were unambiguously observed following IO electrical stimuli. The stimulation serves to both reset the phase of subthreshold oscillation and to entrain a large proportion of neurons to in-phase oscillations. Indeed, synchronization of oscillatory activity over the IO network increased the amplitude of the optical signal to a level that could be detected easily with our imaging set up. Such oscillatory reset was also observed with intracellular recordings from IO neurons (Figures 4, 6). The optically recorded oscillatory clusters have a dynamic spatial organization, and their amplitude depends on the oscillation phase such that they embraced the largest area during the upward phase of the oscillations. Each cluster consisted of a core region and the adjoining area. The core region demonstrated a close to constant size, but the extent of the adjoining area was found to be phase-dependent.

Direct calculation of core and maximum area (i.e., the core region plus the adjoining area at its utmost extent) for several representative clusters gave a mean core area and a mean maximum of several hundred μ2. Because IO clusters are three-dimensional structures, observed in this case as a planar structure indicate that in depth they comprise hundreds of cells. Thus, our optical data indicate that at the network level, the IO nucleus is organized in functionally coupled tridimensional activity clusters. Each cluster is comprised of several hundred cells, which may act in unison to activate groups of thousands of cerebellar PCs simultaneously in agreement with the multiple electrode recordings observed previously.

In conclusion, the dimensions of clusters are probably determined by the IO electrical coupling coefficient, and thus by the magnitude and distribution of the return inhibition from the cerebellar nuclear feedback, which has been demonstrated in previous *in vivo* experiments (Ruigrok and Voogd, 1995; Lang et al., 1996) and supported with mathematical modeling (Leznik et al., 2002; Velarde et al., 2002).

### The climbing fiber conduction isochronicity

From another perspective, while the temporal distribution of activity is well-demonstrated at the olivary level, one may wonder about the time dispersion produced by the olivocerebellar pathway given the different distances between the IO axons and their target PCs. However, if isochronicity is present, then the conduction time between an IO neuron and its PC should be close to uniform and independent of the distance such a signal had to travel. This issue is particularly significant given that the folded nature of such a cortex can increase the path length to the PCs by more than 50%. Furthermore, the correction of the conduction velocity needed to insure synchronicity should be related linearly to distance. This was, in fact, shown to be the case. The time dispersion for a nearly 4 ms conduction time was plus or minus 500 μs to any regions of the cerebellar mantle, regardless of the distance between the IO and the cerebellar cortex at the bottom or top of the of the deep cerebellar folia or at any point in between (Sugihara et al., 1993). The results were based on complex spike latency from 660 different PCs from 12 rats (Figure 8).

Since our original demonstration, this isosynchronicity has been confirmed in further experiments with other cerebellar systems (Ariel, 2005; Brown and Ariel, 2009). A similar finding concerning conduction isochronicity has also been observed in the thalamocortical system and has been interpreted, as in the case of the olivo-cerebellar system, as a mechanism for temporal coherence. In this case, such timing has been related to the temporal coherence associated with cognitive binding (Engel et al., 1997; Salami et al., 2003; Chomiak and PetersHu, 2008; Vicente et al., 2008).

Therefore, the results indicated that the cerebellar cortex, while being deeply folded anatomically behaves, functionally, as an isochronous sphere as far as the olivocerebellar system is concerned. Further, such isochronicity is actually related to the onset time and duration required for proper motor execution (Welsh et al., 1995).

### The olivocerebellar system and error sensing

Finally, the issue of error sensing, which was previously of great interest to cerebellar physiologists, has been treated in detail in excellent reviews concerning IO function (Simpson et al., 1996; De Zeeuw et al., 1998). My personal view is that the error-sensing signal that is often observed in climbing fiber responses—while being a very important functional phenotype—may not be “the central cerebellar function” as some authors claim. From my perspective, the high probability of complex spike activation in relation to unexpected error signals correlates well with such events simply because it is easy to detect. This is the case because climbing fiber activation is massive both when a large reset of the oscillatory phase occurs (Makarenko and Llinás, 1998; Leznik et al., 2002; Chorev et al., 2007; Khosrovani et al., 2007; see also Van Der Giessen et al., 2008 for the connexin 36 role in this large reset), and when a massive temporal reorganization of motor pattern activity is required.

#### Experimental findings

This question was addressed in studies rodent brainstem slices. In agreement with previous intracellular results (Llinás and Yarom, 1986), an extracellular stimulation given at the dorsal border of the IO nucleus generates a full action potential demonstrate that if the cell was oscillating at the time of the stimulus, its oscillations are stopped momentarily, but resumed with a different phase shortly after the stimulation (Llinás et al., 2002). Moreover, in later experiments, it was also determined that such extracellular stimulation may reset the phase without affecting the amplitude or frequency of the subthreshold oscillation (Leznik and Llinás, 2005), and that for most cells recorded, this phase reset could be observed repeatedly with subsequent stimuli (Figure 3). However, the most surprising property discovered was the fact that the oscillation phase shift was remarkably constant and independent of the original phase moment at which the stimulus was delivered.

This constant phase shift is of central importance in defining IO function, as it gives a clear time constraint to the functional states generated by the neuronal ensemble. The reset property of the IO circuit can thus be considered as the main component in the large correction that must be generated when a movement error occurs. This is best illustrated by the fast recovery that we all experience when tripping during locomotion and the fact that we do not fall, while robots do, under similar circumstances.

The issue of error correction has been studied elegantly under conditions where random stimuli require temporal resting under circumstances of robust activation of the cerebellar system (Schweighofer et al., 2004a,b); however, this issue must be addressed further as other views are also clearly present (Gilbert and Thach, 1977; Horn et al., 2004; Catz et al., 2005; Kojima et al., 2010; Popa et al., 2012, 2013). The image one has is of the activation of a very large population of Purkinje cells that mediate a rapid inhibition of the inhibitory cells of the nucleo-olivary pathway, resulting in increased coupling at the olivary level. This event will produce a large and coherent activation of IO neurons; thus, an increased probability of PC complex spike activation ensues. In short, then, error correction is one mode, but not the main mode of IO function.

### Thoughts on the functional significance of two distinct olivocerebellar systems

The rather remarkable differences observed in both the electrophysiology and morphology of these two types of IO neurons clearly implies that the IO nucleus must operate in at least two different modes. While with hindsight we now better understand the problems presented by the lack of oscillatory behavior in the oculomotor system and the presence of physiological tremor in the somato-motor system the findings reported here requires a hypothesis that addresses the necessity of two types of IO neurons in the organization of coordinated motricity.

While the importance of a timing signal has been theoretically assign to the requirements of motor temporal binding (Welsh and Llinás, 1997) by allowing time coherence of motoneuron activation to provide a basic element for motor coordination, a similar case may be made for the oculomotor system. So what would be the difference between somatic and ocular motricity that would require such dramatic functional differentiation? One possible hypothesis relates to the multiple joint organization of the somato-motor system as opposed to the single joint organization of the oculomotor system. In the former case multiple parameters corresponding to different coordinate systems must operate in unison to attain coordination. To this parameter is added muscle feed back that operates in all myotatic reflexes where muscle spindles are simultaneously informing the CNS about the position and rate of movement of each segment of any of our multi-jointed limbs. Because of the tremendous complexity afforded by such massive co-activation of motoneuron pools the temporal requirements become astronomically complicated and a welcome control approach might be to restrict movement to the ballistic properties that we know characterize somato-motor movements (Welsh and Llinás, 1997). By contrast, oculomotor activity does not require the ballistic approach to motor generation since all the parameters are regulated to only one vector in tridimensional space. To this parameter is added the fact that eye movements require a degree of precision not usually demanded of the somato-motor system. Indeed eye movement fixation is only modulated by the microsacadic system that operates at 0.2° in amplitude in an open loop mode. The somatomotor system is far less precise and must operate under conditions where the movement load and momentum vary continuously. For example, as we reach, hold, and lift objects, masticate hard or soft materials, throw a projectile, or return a fast serve with a tennis racket.

In short, we may consider the enormous difference in motor organization as the evolutionary pressure that ultimately determined the motor organization of these two different motor strategies as the root for the very crisp differences in the electrophysiological properties of these two different types of IO neuron.

## Conclusions and implications

Four main issues have been addressed in this short paper concerning the functional organization of the olivo-cerebellar system. (1) The olivocerebellar system seems to be related centrally to the control of motor timing. It's exceptional neuronal characteristics and the network properties that it supports make the olivo-cerbellar system a unique control system, where timing seems to be a central theme. (2) The combination of strong and rather stereotyped intrinsic electrical properties with electrical coupling among the neuronal elements allows the synchronous activation of clusters of neurons. Further, feedback inhibition provides the dynamic variance of the membership of such coupled clusters. (3) The very fundamental property of the resetting of the phase of groups of neurons by a stimulus, such that the new phase is coherent and independent from the original phase, makes this event truly spectacular. (4) When movements require truly continuous control and the issues if multi-joint dynamics are not considered, the IO generates non-oscillatory behavior, as is the case in eye movement kinetics. These four elements give the IO a very powerful set of network properties allowing not only the temporal control of many variables simultaneously, as occurs during motor control, and the possibility of rapid correction in the presence of unexpected events that require rapid global motor correction, but also the possibility of the smooth control that allow eye movement pursue of object displacement in the visual field.

Finally, nature has evolved a mechanism by which this very elaborate cluster dynamic generating system can transmit the timing sequences into a folded cortical geometry, without differential conduction time aberrations, and terminate its path by generating the most powerful synapse in the CNS. If this were not sufficient, the neurons it activates are the largest in the brain, receive just one such climbing fiber afferent, and its output is inhibitory (Ito and Yoshida, 1966). And so, nature has devised one of its most conserved neuronal systems to control motricity by inhibition, a very fitting attribute because it is by selection, via inhibition that the most elaborate neuronal patterns are generated in the CNS.

### Conflict of interest statement

The author declares that the research was conducted in the absence of any commercial or financial relationships that could be construed as a potential conflict of interest.
